# Comparing Clinical Preparedness of Newly Qualified Diagnostic Radiographers Trained With Immersive Virtual Reality vs. Traditional Simulation: A Mixed‐Methods Study

**DOI:** 10.1002/jmrs.882

**Published:** 2025-05-05

**Authors:** Hossein Karimi, Samantha Clarke, Emily Watson

**Affiliations:** ^1^ San Juan de Dios Hospital Santiago Chile

**Keywords:** clinical competence, diagnostic imaging, radiography, radiography education, virtual reality

## Abstract

**Introduction:**

Preparing diagnostic radiographers for clinical roles is vital to ensure readiness for real‐world scenarios. Traditional simulation methods have been central to radiography education, but virtual reality (VR) technology introduces immersive and dynamic environments that may enhance clinical preparedness. This study compares the perceptions of clinical preparedness among newly qualified radiographers trained with VR by Virtual Medical Coaching and traditional simulation methods.

**Methods:**

A mixed‐methods comparative study was conducted with 80 newly qualified radiographers from two universities. Forty were trained using VR, and forty with traditional methods. Participation in the assigned simulation methods was mandatory, but students could opt out of having their responses recorded. All invited students consented to inclusion in the study. Data were collected through semi‐structured interviews, focus groups and objective performance measures, including input from heads of departments. Thematic analysis identified key themes in qualitative data, while quantitative data were analysed using mixed‐effects models, two‐way ANOVA and t‐tests.

**Results:**

The mixed‐effects model showed that VR‐trained students had significantly higher clinical preparedness scores (*β* = 0.905, SE = 0.106, *z* = 8.513, *p* < 0.001). *T*‐tests revealed that VR‐trained students scored higher in confidence, adaptability, technical proficiency and problem‐solving skills (*p* < 0.0001). VR‐trained radiographers also outperformed their traditionally trained counterparts in supervisor evaluations, radiograph quality and emergency performance.

**Conclusion:**

VR training enhances confidence, adaptability and technical proficiency in newly qualified radiographers. Its immersive nature, combined with immediate feedback, contributes to improved clinical preparedness. This study highlights the potential benefits of incorporating VR into radiography education.

## Graphical abstract

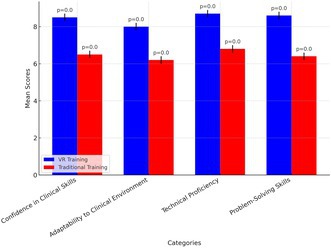

## 1 Introduction

Preparing diagnostic radiographers for their clinical roles is crucial to ensure their readiness for real‐world scenarios. Traditional simulation methods have long been a mainstay in radiography education, providing hands‐on experience with static mannequins and controlled environments. However, the advent of immersive, interactive and dynamic training environments introduces a new dimension that may significantly enhance clinical preparedness. These simulations offer students a more lifelike setting to practice their skills, potentially improving their ability to manage complex and diverse clinical situations.

Clinical preparedness encompasses a radiographer's ability to apply theoretical knowledge, technical skills and critical thinking in patient care. While traditional simulation methods provide valuable experience, they often lack the adaptability needed for complex, evolving scenarios. Immersive simulations, by creating varied and repeatable clinical environments, have the potential to bridge the gap between theoretical learning and practical application, offering a more comprehensive learning experience.

Previous research has shown that radiographers trained with immersive simulation perform better in exams and on clinical placements and report higher levels of course enjoyment compared to those trained with traditional methods [1, 2, 3, 4]. However, the impact on clinical preparedness in the workforce remains underexplored. Despite the increasing adoption of immersive simulation, its impact on the clinical preparedness of newly qualified radiographers remains underexplored. By capturing the experiences of these radiographers, as well as the observations of their heads of departments, this study provides a comprehensive understanding of how different training methods influence clinical readiness.

This study is particularly timely as educational institutions and healthcare providers seek to optimise training methods amidst rapid technological advancements [5].

This study aims to compare the clinical preparedness of newly qualified diagnostic radiographers trained using immersive simulation provided by Virtual Medical Coaching and those trained with traditional methods. By evaluating their clinical performance and preparedness, this research offers evidence‐based insights that contribute to improving radiography education and, ultimately, patient care.

## 2 Methods

### 2.1 Study Design

A mixed‐methods comparative study was conducted to assess clinical preparedness among newly qualified diagnostic radiographers trained with immersive simulations versus traditional simulation methods. This design allowed for the collection of both in‐depth perceptions and objective performance data to comprehensively evaluate training effectiveness [6].

### 2.2 Participants

Eighty radiographers participated, with 40 receiving immersive simulation training using the X‐Ray Pro VR Suite provided by Virtual Medical Coaching and 40 trained using traditional simulation methods. Participants were drawn from two successive cohorts: the first trained with traditional methods, the second with VR simulations. Both groups completed identical clinical scenarios with the same total simulation time, ensuring comparability. The only variable that differed was the training method, with VR replacing traditional physical simulations in the second cohort.

Purposive sampling ensured a diverse range of experiences among participants, selecting individuals based on specific characteristics relevant to the study [7]. All participants were newly qualified radiographers from two universities and had 6 months of clinical experience at the time of the interview. Given that participants did not all start clinical practice at the same time, interviews were scheduled to occur 6 months after each participant's start date. Graduate performance criteria are available in Appendix S1 and S2.

### 2.3 Statistical Analyses

Quantitative data were analysed using three statistical approaches to address different aspects of the research questions:

1. Fixed‐Effects Model—Applied to account for potential university differences while directly estimating the effect of training type on preparedness scores. A fixed‐effects model was chosen over a mixed‐effects model due to the limited number of institutions, which prevented meaningful estimation of random variance. Training type significantly influenced preparedness scores, while university differences were not statistically significant.
2. Two‐Way ANOVA—Used to examine the interaction between ‘Training Type’ (VR vs. traditional) and ‘University’ on preparedness scores. University was treated as a fixed effect to assess within‐study variations.
3. Independent Samples t‐Tests—Used for pairwise comparisons of preparedness metrics (e.g., confidence, adaptability and technical proficiency) between VR‐trained and traditionally trained groups.

Statistical analyses were conducted using Python 3.11, NumPy 1.24 and Pandas 2.0. Descriptive statistics, t‐tests and F‐tests were used to summarise and explore the data. A 10‐point Likert scale was used for supervisor evaluations to provide a more granular assessment of radiographers' confidence, adaptability, technical proficiency and problem‐solving skills [8]. This approach was informed by established assessment frameworks [9]. This scale was selected to distinguish subtle differences in performance, which are crucial in a technical field like radiography [10].

### 2.4 Data Collection Methods

Data were collected through semi‐structured interviews, focus groups and objective clinical performance measures. Interviews and focus groups included radiographers and heads of departments from five hospitals, focusing on perceptions of preparedness, acquired skills, confidence levels and comparative benefits of immersive versus traditional simulation. Focus groups were conducted separately for VR‐trained and traditionally trained radiographers to allow for experience‐based discussions.

Objective performance metrics included: Graduate performance criteria used in assessments are detailed in Appendix S1 and S2.

- Supervisor Evaluation Scores—Based on direct clinical observations of radiographers' competencies.
- Radiograph Quality and Accuracy—Evaluated by a panel of radiologists.
- Performance in Simulated Emergencies—Assessed using standardised emergency simulations involving patient scenarios.

### 2.5 Qualitative Analysis

Thematic analysis, following Braun and Clarke's six‐phase framework, was used to analyse qualitative data. NVivo 14 software facilitated coding and theme development. To enhance validity, triangulation was employed by cross‐verifying data from interviews, focus groups and objective measures. Member checking ensured accuracy by allowing participants to confirm preliminary findings and an audit trail documented the analysis process for transparency. Peer debriefing minimised bias through external review of coding and interpretations.

### 2.6 Bias Minimisation and Ethical Considerations

Blinding of interviewers was not feasible due to the nature of the training methods, but bias was minimised through standardised interview guides and consistent procedures. The researcher conducting the interviews was not involved in clinical supervision or evaluation of participants.

Written informed consent was obtained from all participants. Data collection took place from June to July 2020 and June to July 2021, aligning with the follow‐up period for the two successive cohorts. All data were anonymised and securely stored.

This study received Institutional Review Board approval from the university and each participating hospital under the following references: Universidad de Chile (2018/3465), Hospital Clínico Universidad de Chile (2019/2345), Hospital Sótero del Río (2019/2456), Hospital Dr. Luis Calvo Mackenna (2020/3487), Hospital Barros Luco Trudeau (2020/3512), and Hospital del Salvador (2021/4578, 2021/4601).

## 3 Results

A total of 80 participants were included, with equal representation of VR and traditional training groups.

The VR training included a range of tasks reflecting both routine and complex clinical scenarios. Learners were able to observe the effects of kVp and mAs on image quality, simulating film/screen characteristics before post‐processing was applied. This approach provided insights not typically visible in modern digital radiography (DR), where automatic processing often conceals these variations. By adjusting exposure factors in VR, students developed a deeper understanding of radiographic principles, enhancing their confidence and decision‐making in clinical practice. Key quantitative findings, including supervisor evaluations and performance scores, are summarised in the next section. As shown in Table 1, VR‐trained students achieved significantly higher supervisor evaluation scores compared to traditionally trained students (VR mean = 9.2, traditional mean = 7.4, *t* = 11.235, *p* < 0.0001). While this difference is statistically significant, it also represents a meaningful clinical improvement, as a 1.8‐point increase on a 10‐point scale suggests a noticeable enhancement in real‐world radiographic performance and supervisor‐rated competency.

**TABLE 1:** Summary of fixed‐effects model results.

Coefficient	Estimate	SE	*z*	*p* > |*z*|	[95% CI]
Intercept	6.55	0.012	0	< 0.001	6.52 to 6.58
Training type [T.VR]*	1.97	0.115	0	< 0.001	1.74 to 2.20
University (Fixed effect)	0.0098	0.045	0	0.485	−0.078 to 0.098

* Significant at *p* < 0.001.

### 3.1 Quantitative Findings Overall Preparedness Scores

A fixed‐effects model revealed a significant effect of Training Type on preparedness scores (*β* = 1.97, *p* < 0.001), with VR‐trained students scoring significantly higher than traditionally trained students. The university effect was not statistically significant (*β* = 0.0098, *p* = 0.485), reinforcing the finding that preparedness scores were consistent across institutions. The baseline preparedness score for traditionally trained students was 6.55 (SE = 0.012). These findings confirm that training type, rather than institutional factors, primarily influenced preparedness outcomes. The summary of these results is presented in Table 1.

### 3.2 Effectiveness of Training Type (Two‐Way ANOVA)

A two‐way ANOVA indicated a significant main effect of Training Type on preparedness scores (*p* < 0.001, *η* ^2^ = 0.49). However, no significant main effect of university (*p* = 0.574) or interaction effect between Training Type and University (*p* = 0.111) was found. These findings are detailed in Table 2.

**TABLE 2:** Two‐way anova results comparing training type and university.

Source	df	*F*‐statistic	*p*	Effect size (η^2^)
Training type	1	72.377	< 0.001	0.49
University	1	0.320	0.574	0.01
Interaction effect	1	2.600	0.111	0.03

### 3.3 Category‐Specific Performance

Independent t‐tests showed that VR‐trained students scored significantly higher in all measured categories compared to their traditionally trained peers (*p* < 0.001 for all categories). In addition to statistical significance, the observed differences—ranging from 1.5 to 2.0 points on a 10‐point scale—suggest a clinically meaningful advantage. For example, a 2‐point difference in confidence or adaptability could translate to improved clinical decision‐making and reduced supervision requirements in real‐world practice.

The analysis revealed significant differences in preparedness metrics between VR‐trained and traditionally trained students. VR‐trained students demonstrated higher overall preparedness, confidence, adaptability, technical proficiency and problem‐solving skills. Specifically, the mean preparedness score for VR‐trained students was 8.44, compared to 6.57 for those trained using traditional methods (*t* = 14.96, *p* < 0.0001). Confidence levels were also significantly higher among VR‐trained students (8.48 vs. 6.62, *t* = 14.72, *p* < 0.0001). Adaptability followed a similar trend, with VR‐trained students scoring 8.05 compared to 5.99 in the traditional group (*t* = 12.99, *p* < 0.0001). Technical proficiency was notably enhanced through VR training, with mean scores of 9.0 compared to 7.13 for traditional training (*t* = 10.90, *p* < 0.0001). Lastly, problem‐solving ability was significantly higher in the VR group (8.61 vs. 6.52, *t* = 13.71, *p* < 0.0001). These findings, summarised in Table 3, indicate a substantial advantage for VR‐trained students across all assessed categories.

**TABLE 3:** Comparison of preparedness metrics between training groups (*t*‐test results).

Category	VR mean	Traditional mean	Mean difference	95% CI (lower – upper)	*t*‐statistic	*p*
Preparedness	8.44	6.57	1.87	(1.67–2.07)	14.96	< 0.0001
Confidence	8.48	6.62	1.86	(1.65–2.07)	14.72	< 0.0001
Adaptability	8.05	5.99	2.06	(1.73–2.39)	12.99	< 0.0001
Technical proficiency	9.00	7.13	1.87	(1.54–2.20)	10.90	< 0.0001
Problem‐solving	8.61	6.52	2.09	(1.76–2.42)	13.71	< 0.0001

**FIGURE 1:**
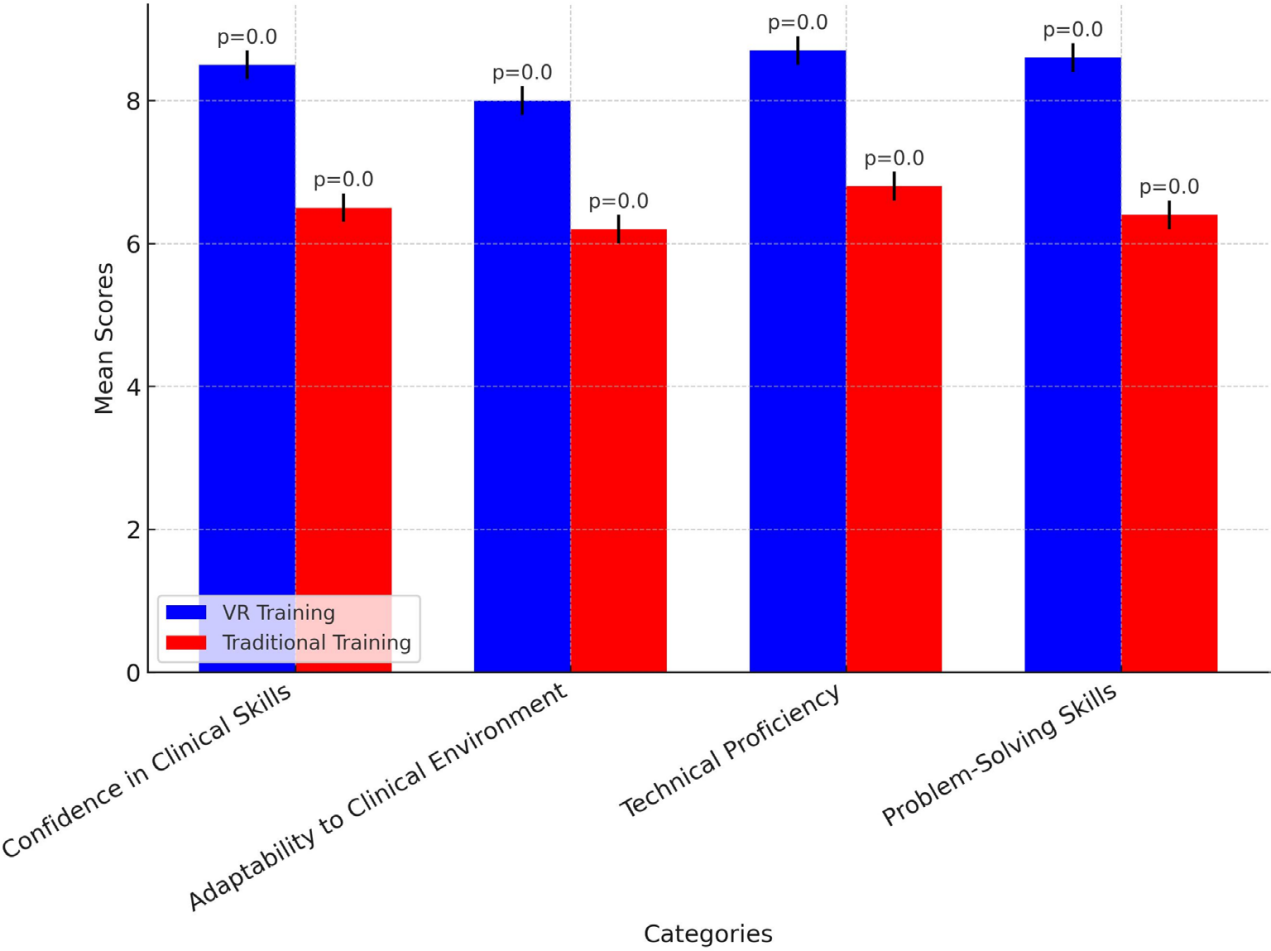
Comparative performance across preparedness categories.

**FIGURE 2:**
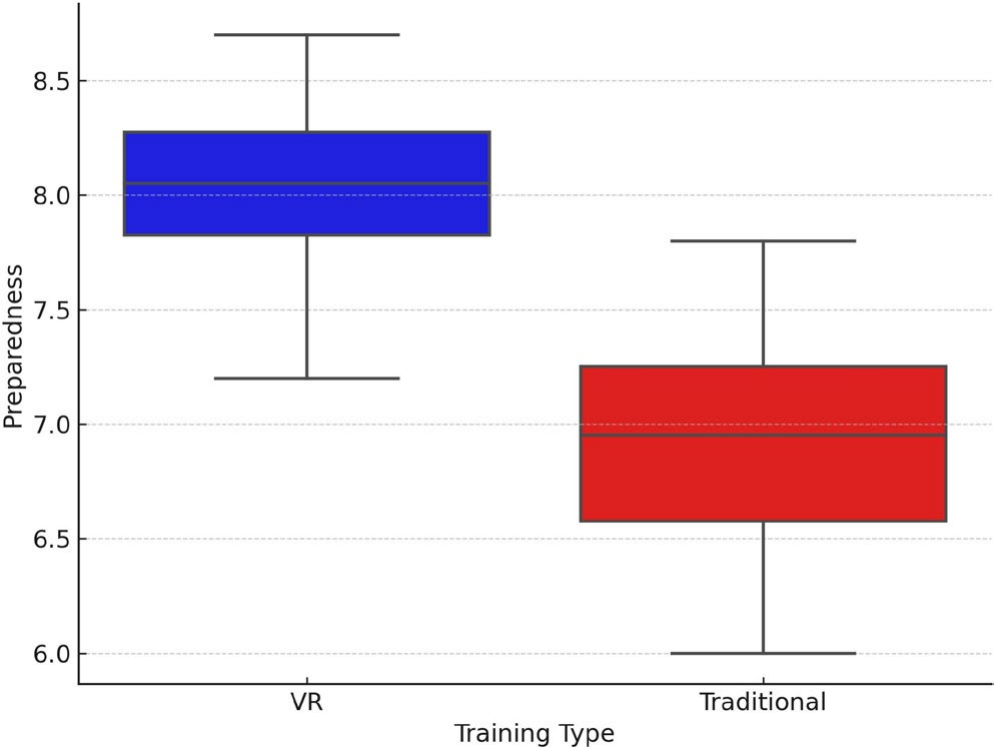
Preparedness scores by training type.

Figures 1 and 2 further illustrate these differences. Figure 1 presents a comparison of mean scores, showing that VR‐trained students consistently outperformed their traditionally trained peers in confidence, adaptability and technical proficiency (*p* < 0.001 for all categories). Figure 2 provides a boxplot of preparedness scores, highlighting that VR‐trained students not only achieved higher median scores but also exhibited lower variability in performance. This suggests that VR training results in more consistent learning outcomes, reinforcing its effectiveness in enhancing clinical preparedness.

Qualitative data collected through thematic analysis reinforced the quantitative findings. VR‐trained radiographers expressed higher confidence in their clinical abilities, noting that the immersive nature of the training helped them adapt more quickly to real‐world settings. Many participants highlighted the value of immediate feedback and advanced scenario‐based training provided by the immersive simulations, which traditional methods often lacked.

The heads of departments who participated in the study corroborated these findings, observing that VR‐trained radiographers demonstrated greater adaptability and technical proficiency in clinical settings. They also noted that these radiographers were more confident and prepared to handle complex patient cases compared to their traditionally trained counterparts. These external evaluations provide an important validation of the self‐reported data from the radiographers themselves and the objective measures obtained from supervisors and radiologists.

Two‐way ANOVA results showed a significant main effect of training type on clinical preparedness (*p* < 0.001), with VR‐trained students scoring higher than their traditionally trained counterparts. No significant main effect of university or interaction effect between university and training type was observed. The two‐way ANOVA results, summarised in Table 2, highlight a significant effect of training type on preparedness scores (*p* < 0.001).

Independent samples *t*‐tests were conducted to compare the scores of VR‐trained and traditionally trained students across various measured categories. The results, presented in Table 3, indicated that VR‐trained students scored significantly higher in all measured categories compared to their traditionally trained counterparts.

## 4 Qualitative Results

Thematic analysis of the qualitative data provided deeper insights into the experiences and perceptions of the radiographers. Key themes and sub‐themes identified from the data are summarised in Table 4.

**TABLE 4:** Key themes from qualitative analysis with supporting quotes.

Theme	Sub‐themes	Example quotes
Perceptions of Clinical Preparedness	Higher Confidence (VR)	‘The VR training made me feel more confident when I started my job’
	Faster Adaptation (VR)	‘I adapted quickly to the clinical environment thanks to VR training’
	Lower Confidence (Traditional)	‘Initially, I was unsure about some procedures’
	Slower Adaptation (Traditional)	‘I needed more initial supervision to adjust to real‐world settings’
Specific Skills and Competencies	Advanced Scenarios (VR)	‘The immediate feedback in VR helped me improve my technical skills quickly’
	Immediate Feedback (VR)	‘The VR scenarios were very realistic and engaging’
	Basic Scenarios (Traditional)	‘The traditional methods provided a good foundation, but I lacked exposure to advanced scenarios’
	Delayed Feedback (Traditional)	‘Feedback in traditional training was often delayed, which hindered immediate learning’
Comparative Benefits and Drawbacks	High Engagement (VR)	‘The VR scenarios were very realistic and engaging’
	Realistic Scenarios (VR)	‘The lifelike VR simulations prepared me well for actual clinical situations’
	Lower Engagement (Traditional)	‘Traditional simulations were helpful but sometimes felt repetitive’
	Less Realistic Scenarios (Traditional)	‘Traditional training lacked some of the realistic scenarios’
Transition to the Workforce	Less Supervision Needed (VR)	‘I required less supervision and adapted faster to the clinical environment thanks to VR training’
	Quicker Adjustment (VR)	‘The VR training prepared me well, and I adjusted quickly’
	More Supervision Needed (Traditional)	‘I needed more initial supervision to adjust to real‐world settings’
	Slower Adjustment (Traditional)	‘It took me a while to adjust to the real‐world settings’
Perceptions of Teaching Methods	Innovative and Effective (VR)	‘Our tutors in the VR training were always looking for the most effective methods to teach us’
		‘The innovative teaching methods in VR greatly enhanced my learning experience’
	Comfortable and Traditional (Traditional)	‘It felt like our tutors in traditional training were just sticking to what they were comfortable with’
		‘Traditional training methods seemed outdated and less effective compared to VR’

Objective measures further validated the qualitative and quantitative findings. The results are summarised in Table 5, following the findings on preparedness, ANOVA results and category‐specific performance.

**TABLE 5:** Comparison of objective clinical performance metrics.

Category	VR mean	Traditional mean	Mean difference	95% CI (lower – upper)	*t*‐statistic	*p*
Supervisor Evaluation Scores	9.2	7.4	1.8	(1.48–2.12)	11.235	< 0.0001
Radiograph Quality and Accuracy	9.1	7.2	1.9	(1.60–2.20)	12.487	< 0.0001
Performance in Simulated Emergencies	8.9	6.8	2.1	(1.78–2.42)	13.004	< 0.0001

Figure 1 illustrates the comparative performance of VR‐trained and traditionally trained students across four key areas: confidence in clinical skills, adaptability to the clinical environment, technical proficiency and problem‐solving skills. VR‐trained students consistently outperformed their traditionally trained peers in all areas, with statistically significant differences (*p* < 0.001). The higher scores in confidence suggest that VR‐trained students felt more assured about their abilities in clinical settings, which is further reflected in their quicker adaptability to clinical environments. This indicates that VR training facilitates a smoother transition from learning to real‐world application. Additionally, the superior scores in technical proficiency among VR‐trained students highlight the benefits of immersive and interactive learning, which allows for repeated practice and immediate feedback. These findings reinforce the effectiveness of VR training in enhancing key competencies required for clinical preparedness.

Figure 2 provides a visual comparison of the preparedness scores between VR‐trained and traditionally trained students. The box plot displays the median, quartiles and potential outliers for each training type, illustrating the distribution and variability of the preparedness scores.

Figure 2 presents a boxplot comparison of preparedness scores. VR‐trained students demonstrated a higher median preparedness score, suggesting greater overall clinical readiness. Both groups exhibited similar variability in scores. These findings support the conclusion that VR training leads to higher and more consistent preparedness levels, as confirmed by both quantitative measures and participant feedback. The results indicate that VR‐trained radiographers achieved higher scores in confidence, adaptability, technical proficiency and problem‐solving skills compared to those trained with traditional methods, as supported by quantitative measures (*p* < 0.001 for all categories).

## 5 Discussion

This study explores the clinical preparedness of newly qualified diagnostic radiographers trained using Virtual Medical Coaching's immersive VR simulations compared to those trained through traditional simulation methods. Clinical preparedness is crucial for radiographers as it determines their ability to apply theoretical knowledge, technical skills and critical thinking in real‐world clinical settings [1, 2, 3]. Traditional simulations have long been a staple in radiography education, but VR introduces a more dynamic, interactive and realistic training environment that may significantly improve readiness and adaptability [11]. This research is particularly timely given the rapid advancements in immersive learning technologies and the need for more effective and efficient educational tools in healthcare [12].

The study demonstrates that VR training provides significant benefits in preparing radiographers for clinical practice. VR‐trained radiographers had significantly higher preparedness scores, enhanced confidence and improved adaptability compared to their traditionally trained counterparts. The immersive nature of VR, coupled with real‐time feedback, created a more engaging and realistic training experience, better‐preparing radiographers for the complexities of clinical practice, as also demonstrated by Grewe [13].

This enhanced confidence is reflected in their quicker Adaptability to Clinical Environments, suggesting that VR training facilitates a smoother transition from learning to real‐world application. Additionally, the higher scores in technical proficiency among VR‐trained students demonstrate their superior technical skills, likely due to the immersive and interactive nature of VR training, which allows for repeated practice and immediate feedback. These findings underscore the potential of VR training to better prepare students for clinical practice.

These findings bridge the gap between theoretical learning and practical application in radiography education.

Quantitative results showed that VR‐trained radiographers consistently outperformed their peers across all measured categories, including confidence, adaptability, technical proficiency and problem‐solving skills. These findings are reinforced by the qualitative data, which revealed that VR‐trained students valued the ability to practice advanced scenarios and receive immediate feedback. Heads of departments corroborated these findings, observing that VR‐trained radiographers demonstrated superior technical proficiency, confidence and adaptability. These external evaluations validate both the objective measures and the self‐reported perceptions, highlighting VR's effectiveness in enhancing technical and decision‐making skills [14, 15, 16, 17].

One of the most striking aspects of the findings is the standardisation that VR training offers. All learners experienced the same scenarios and challenges, ensuring uniformity in training outcomes. In contrast, traditional training can vary depending on whether students actively participate in simulations or observe from the sidelines. This variability can lead to unequal learning experiences, as students who observe may not develop the same hands‐on proficiency or decision‐making skills as those directly involved in the simulation. By eliminating such discrepancies, VR provides each learner with equal opportunities to refine their skills, leading to better patient care and fewer errors in clinical practice [18, 19, 20].

The lack of significant variability between universities in statistical analyses suggests that the effectiveness of VR training is not institution‐specific. However, given the sample size, we acknowledge that smaller institutional differences may not have been detectable. While this suggests that training type had a dominant effect in this study, this does not confirm that the findings generalise across all institutions. ANOVA is not highly powered to detect smaller interaction effects, particularly with a sample size of 80 participants. Therefore, institution‐specific variations may exist but were not detected due to sample size constraints. Further research with a larger sample across multiple institutions is needed to assess the broader applicability of these findings. Despite this, the observed improvements in preparedness scores indicate a robust potential for VR training to enhance clinical readiness across different settings. This consistency indicates that the benefits of VR training are not institution‐specific and can be generalised across diverse educational settings. This scalability is essential for broader implementation, ensuring equal opportunities for learners and consistent improvements in preparedness and patient care [21, 22].

In addition to educational benefits, VR training provides operational advantages when radiographers enter clinical practice. The higher preparedness of VR‐trained radiographers reduces the need for extended supervision, allowing senior staff to focus on patient care and advanced procedures. This accelerated transition to independent work can also minimise onboarding costs and staffing burdens, offering financial and efficiency benefits to healthcare institutions [15, 16, 17, 23, 24]. Furthermore, the ability to onboard new radiographers efficiently without compromising care quality helps healthcare institutions address staffing shortages and maintain productivity. This efficiency could reduce reliance on temporary staff and overtime, providing both financial and operational benefits [18, 25].

The objective measures of clinical performance, including supervisor evaluation scores, radiograph quality and accuracy and performance in simulated emergencies, provided strong validation for these perceived benefits. VR‐trained radiographers consistently demonstrated higher performance in all these areas, reinforcing the notion that immersive VR simulations not only enhance confidence but also translate into superior clinical performance. These findings are supported by studies showing that VR‐based training improves both theoretical knowledge and practical skills [26, 27, 28, 29].

VR training also offers distinct advantages in professional development. The ability to practice complex procedures in a safe, controlled environment allows learners to refine their technical and decision‐making skills without fear of real‐world consequences. These advanced scenario‐based learning opportunities are particularly valuable in preparing radiographers to handle complex cases from the outset of their careers. The findings suggest that VR training has the potential to transform radiography education by providing a more engaging, realistic and effective learning experience, ultimately producing healthcare professionals who are better prepared to meet the demands of modern clinical practice [18, 19, 20].

## 6 Limitations and Future Research

While this study provides strong evidence for the benefits of VR training, several limitations should be acknowledged. The sample size, while adequate for this analysis, could be expanded in future studies to enhance generalisability. Additionally, multi‐centre trials could provide more comprehensive insights into the variability of VR training effects across different contexts. Future research should also investigate the long‐term impact of VR training on clinical performance, decision making, teamwork and career progression.

## 7 Conclusion

This study demonstrates the significant advantages of VR training in enhancing the clinical preparedness of newly qualified diagnostic radiographers. VR‐trained students reported higher confidence, faster adaptation and better technical skills compared to their traditionally trained peers. These findings, supported by objective measures, underscore the effectiveness of VR training. Incorporating immersive VR training into radiography education offers a scalable, standardised and cost‐effective solution for training healthcare professionals. As VR technology evolves, its integration into medical education holds great promise for improving training outcomes and enhancing patient care.

## Ethics Statement

This study received Institutional Review Board approval from the universities and each participating hospital under the following references: 2019/2345, 2019/2456, 2020/3487, 2020/3512, 2021/4578 and 2021/4601.

## Consent

Written informed consent was obtained from all participants. Data were anonymised and securely stored and participants were informed that their participation was voluntary and that they could withdraw at any time without any consequences.

## Conflicts of Interest

The authors declare no conflicts of interest.

## Supporting information


Appendix S1.

Appendix S2.

Structured Interview Guide
[Correction added on 17 September 2025, after first online publication: The ‘’Structured Interview Guide’’ has been uploaded as additional supplement information file in online version.]

## Data Availability

The data supporting the findings of this study are available from the corresponding author upon reasonable request.
