# Supplementary material for: Comparing Clinical Preparedness of Newly Qualified Diagnostic Radiographers Trained With Immersive Virtual Reality vs. Traditional Simulation: A Mixed‐Methods Study
Source: J Med Radiat Sci. 2025 May 5;72(Suppl 2):S70–8. doi: 10.1002/jmrs.882 (PMC12449592; doi:10.1002/jmrs.882)
Supplement: Supplementary file 1 — Appendix S1. [file JMRS-72-S70-s001.docx]

**Appendix 1: Graduate Performance Criteria for Radiography Graduates in University A.**

The performance criteria for radiography graduates focus on core competencies, practical skills, research methodologies, ethics, and professional development. These criteria align with national and international standards to ensure that graduates are prepared for clinical, academic, and research environments.

**1. Core Clinical Competencies**

- **Radiographic Imaging Techniques:**
  Ability to accurately perform routine and specialized radiographic procedures using appropriate protocols and technologies.
  - Correct positioning of patients for optimal imaging.
  - Understanding the principles of radiation physics and safety.
  - Knowledge of imaging modalities such as MRI, CT, ultrasound, and interventional radiology.
- **Radiation Safety and Protection:**
  Application of radiation protection principles to safeguard patients, staff, and oneself from unnecessary exposure.
  - Use of lead shielding, dosimeters, and adherence to ALARA (As Low As Reasonably Achievable) principles.
  - Proper handling of radiation-emitting equipment.
- **Patient Care and Communication:**
  Provision of high-quality patient care and the ability to communicate effectively with diverse populations.
  - Clear explanation of procedures to patients.
  - Compassionate and culturally sensitive care.
  - Ability to assess and respond to patient needs and emergencies.

**2. Professional and Ethical Conduct**

- **Ethical Standards and Legal Responsibility:**
  Understanding and application of ethical standards and legal responsibilities in radiographic practice.
  - Adherence to the **Código de Ética Radiológica** in Chile.
  - Confidentiality of patient records and images in accordance with national laws.
- **Medical Ethics:**
  Demonstration of understanding of key medical ethical principles, such as autonomy, beneficence, non-maleficence, and justice.
  - Ethical decision-making in complex situations (e.g., informed consent for imaging procedures).
  - Awareness of the ethical implications of emerging technologies and radiography practices.

**3. Technical and Analytical Skills**

- **Image Quality Evaluation:**
  Ability to assess the quality of radiographic images and make necessary adjustments to ensure diagnostic accuracy.
  - Identifying artifacts or errors and rectifying them.
  - Knowledge of image post-processing techniques.
- **Equipment Operation and Troubleshooting:**
  Proficiency in the operation and basic troubleshooting of radiographic equipment.
  - Routine equipment checks and calibration.
  - Reporting and addressing equipment malfunctions promptly.

**4. Collaborative and Interdisciplinary Practice**

- **Teamwork in Healthcare Settings:**
  Effective collaboration with multidisciplinary teams, including physicians, nurses, and other healthcare professionals.
  - Active participation in team meetings and case discussions.
  - Contribution to patient management plans, particularly regarding imaging needs.
- **Research and Evidence-Based Practice:**
  Application of evidence-based practice in radiography, including participation in or understanding of relevant research.
  - Ability to critically appraise scientific literature and apply findings to clinical practice.
  - Participation in quality improvement initiatives within the department.

**5. Research Methods and Research Ethics**

- **Research Methods:**
  Competence in applying research methodologies relevant to radiography and medical imaging.
  - Designing research projects, including quantitative and qualitative research designs.
  - Data collection, statistical analysis, and interpretation of results.
- **Research Ethics:**
  Understanding and application of ethical principles in conducting research.
  - Familiarity with ethical guidelines for human subjects in research.
  - Ensuring informed consent, confidentiality, and the protection of vulnerable populations in research.
  - Awareness of ethical issues related to research misconduct, including plagiarism and data fabrication.

**6. Specialized Knowledge Areas**

- **Advanced Imaging Techniques:**
  Understanding and application of advanced imaging techniques where applicable (e.g., PET, nuclear medicine).
  - Training in specialty imaging procedures, including interventional radiology and radiation therapy.
- **Information Technology in Radiography:**
  Competence in the use of PACS (Picture Archiving and Communication System) and other digital imaging systems.
  - Efficient use of electronic medical records and data security protocols.

**7. Cultural Competence in Healthcare**

- **Cultural Sensitivity and Inclusivity:**
  Ability to deliver radiography services in a culturally competent manner, considering the diverse population of Chile.
  - Awareness of indigenous health needs, particularly among Mapuche and other local communities.
  - Customizing patient communication and care based on cultural values and norms.
