# Supplementary material for: Comparing Clinical Preparedness of Newly Qualified Diagnostic Radiographers Trained With Immersive Virtual Reality vs. Traditional Simulation: A Mixed‐Methods Study
Source: J Med Radiat Sci. 2025 May 5;72(Suppl 2):S70–8. doi: 10.1002/jmrs.882 (PMC12449592; doi:10.1002/jmrs.882)
Supplement: Supplementary file 2 — Appendix S2. [file JMRS-72-S70-s003.docx]

**Appendix 2: Graduate Performance Criteria for Radiography Graduates in University B**

The performance criteria for radiography graduates at University B focus on key clinical competencies, practical skills, research proficiency, and ethical practice. These criteria ensure that graduates meet the standards expected in clinical, research, and academic environments, both nationally and internationally.

**1. Core Clinical Competencies**

- **Radiographic Imaging Techniques:**
  Graduates must demonstrate competence in performing a wide range of radiographic procedures, adhering to the university's protocols and current clinical guidelines.
  - Proficiency in patient positioning for optimal imaging.
  - Understanding of radiation physics, including attenuation and image formation.
  - Familiarity with various imaging modalities such as MRI, CT, and ultrasound.
- **Radiation Safety and Protection:**
  Strict adherence to radiation safety practices, prioritizing the protection of patients, colleagues, and oneself.
  - Efficient use of radiation shielding and monitoring tools (e.g., dosimeters).
  - Implementation of ALARA principles in daily practice.
- **Patient Care and Communication:**
  Strong patient-centered communication skills, ensuring clarity and empathy in interactions with diverse populations.
  - Providing clear instructions and comfort to patients before, during, and after procedures.
  - Ability to respond effectively to patient needs, including emergencies.

**2. Professional and Ethical Conduct**

- **Ethical Standards and Legal Responsibility:**
  Graduates are expected to uphold high ethical standards in radiographic practice, adhering to both local and global ethical frameworks.
  - Compliance with legal regulations concerning patient privacy and data security, including adherence to University B’s own ethical code.
  - Maintaining professionalism in handling sensitive information, both written and digital.
- **Medical Ethics:**
  Knowledge and application of fundamental medical ethics, including respect for patient autonomy and well-being.
  - Informed consent processes in imaging.
  - Decision-making in ethically complex cases, particularly around emerging radiographic technologies.

**3. Technical and Analytical Skills**

- **Image Quality Evaluation:**
  Ability to critically assess radiographic images for quality and diagnostic accuracy, and make necessary adjustments when needed.
  - Identifying common image artifacts and applying corrective actions.
  - Knowledge of advanced image processing techniques to enhance diagnostic output.
- **Equipment Operation and Maintenance:**
  Proficiency in the operation and routine maintenance of radiographic equipment.
  - Reporting and resolving equipment issues promptly.
  - Ensuring proper calibration of devices as per clinical guidelines.

**4. Collaborative and Interdisciplinary Practice**

- **Teamwork in Healthcare Settings:**
  Effective collaboration with healthcare teams, contributing meaningfully to patient care and radiological decision-making processes.
  - Active involvement in interdisciplinary discussions regarding imaging needs and outcomes.
  - Working efficiently within a healthcare team, demonstrating respect and consideration for the roles of other professionals.
- **Research and Evidence-Based Practice:**
  Application of evidence-based practice, integrating research findings into clinical decision-making.
  - Ability to review and critically appraise academic literature relevant to radiography.
  - Applying research findings to enhance clinical practice and patient care.

**5. Research Methods and Research Ethics**

- **Research Methods:**
  Understanding and application of fundamental research methodologies, with an emphasis on those relevant to radiography.
  - Ability to design and conduct research, incorporating both quantitative and qualitative methods.
  - Proficiency in data collection, analysis, and interpretation using statistical tools relevant to medical imaging.
- **Research Ethics:**
  Comprehensive understanding of research ethics, with a focus on responsible conduct in radiography-related research.
  - Adherence to ethical principles concerning human subjects research.
  - Ensuring patient privacy, informed consent, and integrity in all research activities.
  - Awareness of the ethical issues surrounding the use of patient data in research.

**6. Specialized Knowledge Areas**

- **Advanced Imaging Techniques:**
  Proficiency in advanced imaging techniques such as nuclear medicine and PET, with the ability to apply these techniques in clinical practice.
  - Knowledge of specialized radiographic procedures, including interventional radiology.
  - Keeping up with technological advancements and innovations in imaging techniques.
- **Health Informatics and Radiography:**
  Competence in using health informatics systems for the effective management of radiographic images and patient records.
  - Proficiency in the use of PACS (Picture Archiving and Communication System) and other medical imaging software.
  - Awareness of cybersecurity protocols in the handling of digital health records.

**7. Cultural Competence in Healthcare**

- **Cultural Sensitivity and Inclusivity:**
  Demonstration of cultural awareness and sensitivity in the provision of radiography services to a diverse population.
  - Tailoring communication and care to the specific cultural, social, and linguistic needs of patients.
  - Consideration of the health beliefs and practices of local communities and indigenous populations.
