# Supplementary material for: Comparing Clinical Preparedness of Newly Qualified Diagnostic Radiographers Trained With Immersive Virtual Reality vs. Traditional Simulation: A Mixed‐Methods Study
Source: J Med Radiat Sci. 2025 May 5;72(Suppl 2):S70–8. doi: 10.1002/jmrs.882 (PMC12449592; doi:10.1002/jmrs.882)
Supplement: Supplementary file 3 — Structured Interview Guide [Correction added on 17 September 2025, after first online publication: The ‘’Structured Interview Guide’’ has been uploaded as additional supplement information file in online version.] [file JMRS-72-S70-s002.pdf]

Structured Interview Guide

Qualitative Phase. VR vs. Traditional Simulation Study

Interviewer Notes (Not read aloud):
Interviews were conducted via Zoom or in person in a quiet space.
Participants were interviewed within 4 to 6 weeks of completing their clinical placement.
Interviews lasted approximately 30 to 40 minutes.
Interviews were audio recorded and transcribed verbatim.
NVivo 14 was used for coding and analysis.
The interviewer was not involved in participants' assessment or supervision.

Introduction (Read aloud to participant):
"Thank you for agreeing to take part. This interview is about your experience of transitioning from training into clinical practice. We're interested in what helped you feel prepared, or what did not. Everything you say will be confidential and anonymous in the final report. You're free to skip any question or stop the interview at any time."

Section 1: Clinical Preparedness and Emotional Readiness

When you first started clinical work, how prepared did you feel overall?
Prompt: Can you describe what gave you that impression?
Were there any parts of your training that felt particularly helpful or unhelpful in preparing you?

Section 2: Confidence and Independent Practice

How confident did you feel when performing procedures on your own?
Prompt: Were there any moments that made you question or reinforce your confidence?

Section 3: Technical Skill and Equipment Use

How prepared did you feel in terms of using the equipment and selecting exposures?
Prompt: Did you rely on preset protocols, or did you adjust settings independently?
Can you describe a moment when you had to problem-solve during a scan or positioning?
Prompt: What did you fall back on: your training, intuition, or help from others?

Section 4: Real World Adaptation

Did your training help you adjust to the clinical environment, such as workflows, pace, or communication?
Were there any surprises in your clinical placement that were not covered in training?

Section 5: Emergencies or Unusual Situations

Did you experience any urgent or unpredictable clinical scenarios (for example, fainting or trauma cases)?
How confident were you in responding, and did anything in your training help?

Section 6: Feedback and Learning Loop

What kind of feedback did your supervisors or mentors give you during placement?
Prompt: Did they comment on how prepared or unprepared you seemed?
Were there any situations where you felt you underperformed? If so, what would have helped?

Section 7: Reflective Insight

Looking back, what would you change about your training to improve real world readiness?

Is there anything else you want to share that might help us understand your transition to practice?
